# Statistical modeling of the equine third metacarpal bone incorporating morphology and bone mineral density

**DOI:** 10.1371/journal.pone.0194406

**Published:** 2018-06-06

**Authors:** Helen Liley, Ju Zhang, Elwyn C. Firth, Justin W. Fernandez, Thor F. Besier

**Affiliations:** 1 Auckland Bioengineering Institute, University of Auckland, Auckland, New Zealand; 2 Department of Exercise Sciences, University of Auckland, Auckland, New Zealand; 3 Department of Engineering Science, University of Auckland, Auckland, New Zealand; Massey University, NEW ZEALAND

## Abstract

The objective of this study was to describe the three-dimensional shape and subchondral bone mineral density (BMD) variation of the equine distal third metacarpal bone (MC3) using a statistical shape model. The association between form and function builds upon previous two-dimensional observations of MC3 epiphyseal structure. It was expected that the main source of variation would be an increase in overall MC3 bone size, correlated to an increase in subchondral BMD. Geometry and bone mineral density was obtained from CT image data of 40 healthy Thoroughbred horses. This was used to create a statistical shape model, in which the first ten components described 75% of the variation in geometry and BMD. The first principal component described an increase in overall size of the MC3 distal epiphysis, coupled with higher BMD on the disto-palmar and dorso-proximal surfaces. The second component was qualitatively described as an increased convexity of the sagittal ridge at the dorsal junction of the epiphysis and the metaphysis, coupled to increased BMD in that region. The third component showed an increase in lateral condylar surface area relative to medial condylar area. As the condyle reduced in relative surface area, the BMD at both dorsal condyles increased. The statistical shape analysis produced a compact description of 3-D shape and sub-chondral bone mineral density variation for the third metacarpal bone. This study uniquely illustrates the shape variations in a sample population of MC3 bones, and the corresponding changes in subchondral BMD.

## Introduction

The equine metacarpo-phalangeal (fetlock) joint is the most common site of injury in the forelimb of the Thoroughbred racehorse [1–3]. Lateral condylar fracture of the distal third metacarpal (MC3) or metatarsal (MT3) bone is the most common fatal fracture site for all types of race [4]. Most condylar fractures occur as an endpoint of stress-induced bone adaptation due to cyclic, cumulative loading [5–8]. The fetlock joint is susceptible to fracture due to its relatively small articulating surface area and the high magnitude of contact forces experienced during galloping [9–11] Research on human joint mechanics has shown that stress distribution and fracture risk are affected by both bone size and epiphyseal bone geometry [12–14]. However, the relationship between equine MC3 morphology (size and geometry) and site-specific bone adaptation leading to fracture requires further investigation.

Morphological observations such as increased asymmetry between the medial and lateral MC3 condyle [15] and a decreased prominence of the sagittal ridge [16] have been associated with increased risk of MC3 condylar fracture. However, these studies did not correlate morphological findings with material properties in the subchondral bone. Subchondral bone mineral density affects mechanical stress distribution through the tissue, and a significant increase in bone mineral density (BMD) occurs in the distal epiphysis of MC3 with age and exercise [17]. Using qualitative observations, the spatial distribution of BMD has been used to explain fracture initiation and propagation in equine MC3 condyles [1, 17–20].

Condylar fatigue fractures occurring in the equine MC3 bone are consistent in their configuration [8]. Lateral condylar fractures initiate at the parasagittal groove, and propagate through previously modeled bone. There is some evidence of focal porosity in the parasagittal groove contributing to fracture propagation [21]. Again, the influence of how size and shape contribute to these observations is not known, despite both being important contributors to mechanical stress in highly loaded mammalian joints.

Information on variation in the geometry of the equine MC3 epiphysis is sparse, because of the difficulty of making consistent and meaningful measurements of complex shapes. Most of the previous research presents two-dimensional scalar results, which are dependent on the choice of landmarks, and the angle and consistency of plain film radiographs [22]. A method to accurately quantify the three-dimensional shape variation in a population is statistical shape modelling [23, 24]. Statistical shape modeling commonly uses principal component analysis (PCA) to decompose shapes into a set of statistically significant components, which represent the axes along which most shape variation occurs [25]. This type of analysis is useful for quantifying gross morphological changes [26, 27], as well as local shape changes in specific regions of interest, such as the sagittal ridge of the third metacarpal bone.

In this analysis, a statistical shape model is used to capture the three-dimensional morphological variation in a sample of computed-tomography (CT)-scanned MC3 bones (n = 40), by establishing point-to-point correspondence between each segmented bone. Bone mineral density for the subchondral bone is incorporated using a point distribution model [28] to investigate the relationship between bone shape and bone mineral density. The model uses PCA to characterize the principal components of shape and BMD variation [24, 28].The first objective of this study was to describe the shape and subchondral BMD variation of the equine distal MC3 bone using a statistical shape model. The second objective was to use this model to investigate whether the form-function relationships shown in the model reflect existing observations of epiphyseal structure, and to investigate if there are other components or geometric features that contribute to total sample variability. It is anticipated that the main source of variation would be a size-scaling of the bone, and that an increase in size would be correlated to an increase in subchondral BMD.

## Materials and methods

### Overview

**Fig 1:**
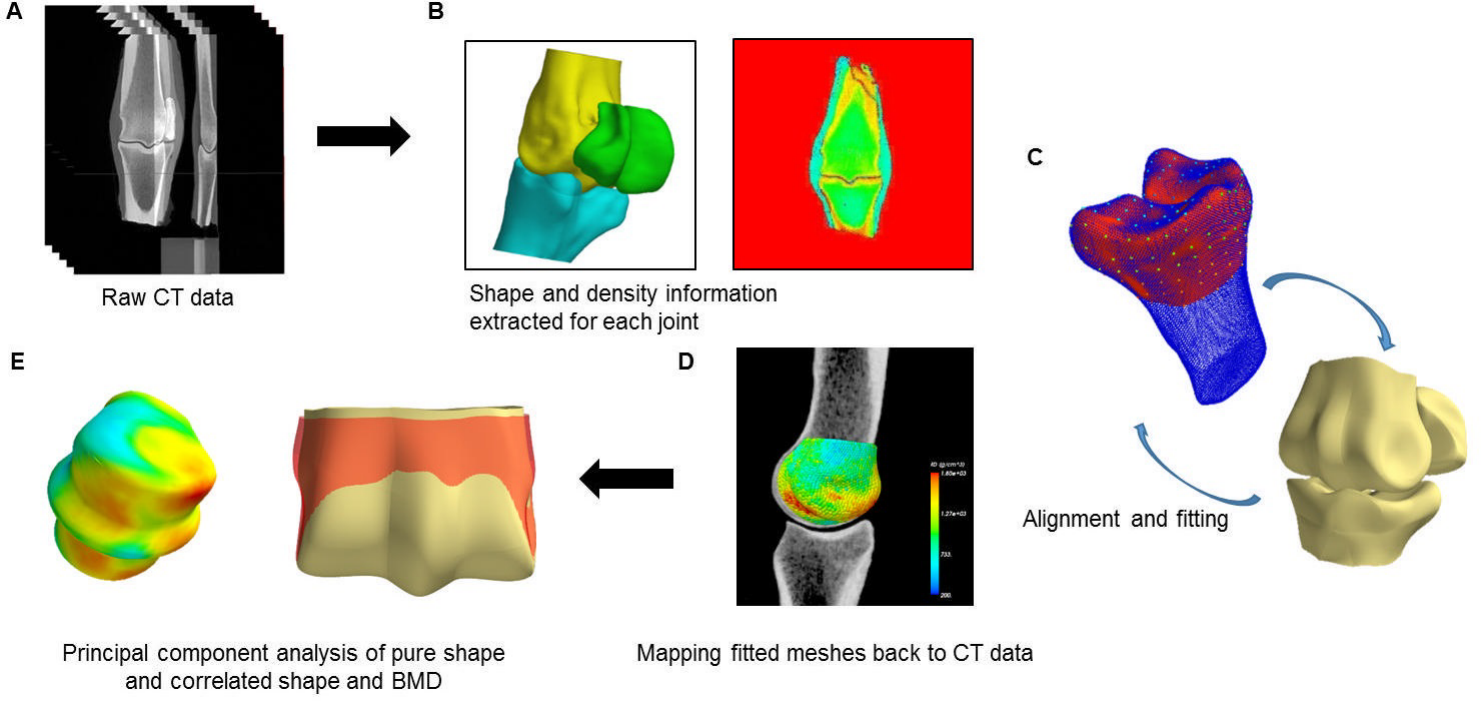
Workflow to generate a statistical shape model of the MC3 bone using CT imaging data.

To understand the variation in the morphology and bone mineral density of the MC3 across the population, a statistical model was trained using CT scans collected from forty mature Thoroughbred horses. The development of the statistical shape model was based on the methods of Zhang et al [24]. This required establishing correspondence between each specimen so that the location and bone mineral density at any given point in one model could be related to an equivalent point in another model. The workflow describing these steps is illustrated in Fig 1 and described below.

### Specimens

The MC3 bones used in this study were from forty mature Thoroughbred horses. The horses were sent to the abattoir for reasons assumed to be unrelated to metacarpo-phalangeal joint injury. This assumption was based off the requirements of the Code of Welfare for transport within New Zealand, which prohibits any animal from travelling if it is displaying signs of injury, disease or physical abnormality (all specimens were transported to the facility the previous day).

Limited information on the horses was obtained from the brands on the shoulder of the animals, but this was unable to be traced, or not visible, for more than 50% of the horses. From the known information, racing history was traceable for twelve of the horses, and a further five were known to be unraced. Twenty-six of the horses were female, and fourteen were male. Eleven horses were younger than four years of age (mean 3.36 ± 0.8 years), and twenty-two were older than four years of age (mean 8.82 ± 4.2 years).

#### A: Raw CT data: Imaging protocol

The left metacarpo-phalangeal joints were transected at the junction of proximal and distal half of both the MC3 and proximal phalangeal bone, labeled, and frozen (-20°C) prior to being transported to the imaging facility. The MC3 bones were scanned using a Siemens SOMATOM CT scanner (140 KV; in plane resolution 0.3 mm, slice thickness 0.6 mm). In the same image space, a hydroxyapatite (HA) phantom of known mineral density of 800mg/mm^3^ was included in each image.

The CT images were examined for bone pathology, and no obvious damage or lesions were found in the specimens used for the study.

#### B: Shape and density information extraction

Data clouds representing the surfaces of the left MC3 bones were segmented from the CT images using Stradwin (Cambridge University, Cambridge, UK), and constitute the sample population, or “training set”.

#### C: Alignment and fitting

From the training set, one data cloud was chosen as the template. The choice of template mesh was based on qualitative examination of the bones, in which the chosen specimen appeared to be representative of the other horses in terms of size and shape, and had no obvious geometric deformities. A custom template cubic Lagrange, piece-wise parametric mesh was created to closely fit the surface of the template data cloud. The proximal cut off of the mesh was 45 mm proximal to the most distal point on the MC3. This was chosen due to the variable lengths of the transected MC3 bones, and included the epiphysis and part of the metaphysis [29]. This mesh was subsequently registered (<0.5 mm RMS) with each data cloud using host mesh fitting [30]. Following this, a rigid alignment minimized the least squares distances of corresponding points on each fitted mesh, such that PCA could be performed on the node coordinates to obtain a shape model [24, 30].

The mean mesh of the shape model was then refitted to each data cloud, this time using the shape model constraint to propagate correspondence. A new PCA shape model was then trained on the refitted meshes. This fitting and training process was repeated until the RMS was reduced to <0.3 mm and did not continue to decrease with subsequent fitting. Finally, a principal component analysis (see *Sub-heading E*. below) on the maximally correspondent meshes yielded the principal components of variation in the bone.

#### Section C.1 Morphometry analysis

**Fig 2:**
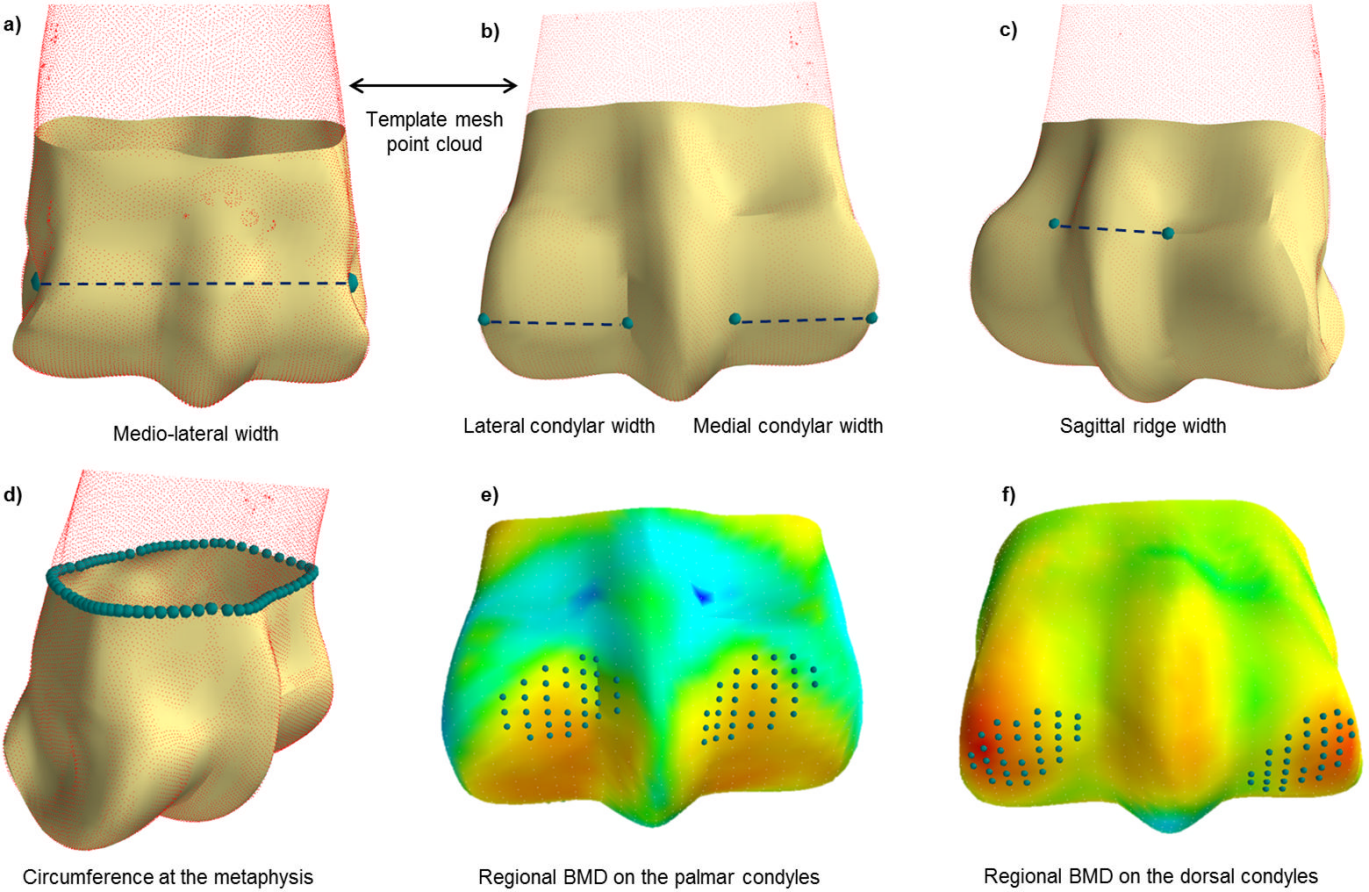
Initial estimates of third metacarpal bone shape and parameters to quantify changes observed in the principal component analysis, the segmented point cloud from the original CT data is included as a reference. Large blue points indicate anatomical landmarks identified from a template mesh and subsequently fit to the population to measure morphological parameters.

Landmark nodes on the template 3D mesh were manually identified (Fig 2) and used to calculate MC3 geometric features following mesh fitting across the population. Since the template mesh was consistent and fit to the segmented point clouds for each specimen, the landmark nodes were automatically obtained for each fitted mesh. The use of a template mesh allowed consistent definition of features by these landmark nodes (indicated by the large points in Fig 2A–2D). The following features were quantified: medio-lateral width, lateral condylar width, medial condylar width, sagittal ridge width, metaphysis circumference. In addition, nodes were selected in the palmar and dorsal condylar regions to determine average subchondral BMD (see D below; Fig 2E–2F).

#### D: Mapping fitted meshes back to CT data

Once the surface mesh had been fitted to each segmented point cloud, it was mapped back to the original DICOM files using a custom Python script. At each node location, a vector normal to the surface was projected inwards 5 mm, which is the approximate depth at which the porosity of the subchondral bone plate increases in mature racehorses [31]. The CT data was sampled along this vector, and the average value (Hounsfield Units) was assigned to the node.

Greyscale values were converted from Hounsfield units to apparent bone mineral density with the following equation:

$$\rho_{HA}=\frac{CT_{bone}-CT_{H_{2}O}}{CT_{HA}-CT_{H_{2}O}}\times \rho_{phantom}$$ (1)

The empiric relationship described by Dalstra et al, and McKellop [32,33] was used to calculated the moduli:

$$\rho_{app}=\frac{\rho_{HA}}{0.626},\;E=2017.3\rho_{app}^{2.46}$$ (2)

Where ρ_app_ is the apparent bone mineral density (g.cm^-3^) and ρ_HA_ the HA equivalent density calculated from Eq (1).

#### E: Principal component analysis of shape and bone mineral density

The correspondence between every bone in the training set enabled direct relation between the location and BMD at any given point in the model to a correspondent point in another model.

PCA provided a statistical technique to decompose the data in the training set into its significant components. The training dataset formed an N × 3n matrix, where N is the number of geometries in the training set and n is the number of nodes in the mesh. Singular value decomposition was used to decompose N into its principal components [34]. This PCA model allowed any shape (x) in the training set to be approximated as a sum of the mean shape ($\overset{-}{x}$) and the weighted sum of n principal components (ϕ) [24].

$$x=\bar{x}+\overset{}{\underset{}{\sum_{i=0}^{n}}}\omega_{i}\phi_{i}$$ 3

In the model (3) above, to create a new bone instance **x**, the mean of the training geometries $\overset{-}{x}$ was summed, with the first *n* components weighted by scores ***ω*_*i*_**. *n* was chosen such that the cumulative variance explained by the components accounted for 80% of the total variation in the population. Singular value decomposition was used to reduce the size of the correlation matrix [34].

To incorporate bone mineral density, a point distribution model was used. In this case the training dataset was an N × 4n matrix, containing dimensional data x, y, and z with modulus data I [28, 35]. A correlation-based PCA approach was used, due to the data containing mixed units [36]. This PCA model was able to examine the correlated effect of each principal component of variation on shape and spatial variability of bone density.

### Quantitative and qualitative analysis

For each principal component of variation, the bone shape was reconstructed at ±2 standard deviations, to represent 95% of the population. Metrics including the total area, the condylar area and the medio-lateral width (Fig 2) were compared to literature and used to quantitatively describe the variation in the first three principal components.

In the qualitative assessment, each component was viewed in isolation over the range of ±2 standard deviations of the mean.

## Results

### Principal component analysis

In the correlated point distribution model, the first principal component described 30% of the total variation. This was quantitatively assessed as a 30% increase in total area, with a 10–20% average increase in BMD density on the palmar and dorsal condyles (S1 Table). The second component described 9% of the variation, and was qualitatively assessed as increased ridge prominence on the dorso-proximal aspect, coupled to increased BMD in that region. The observed change was most obvious on the dorsal side, proximal to the circular curvature of the distal epiphysis. The third mode, accounting for 7% of the total variation, showed a 10% increase in lateral condylar area relative to medial condylar area, which was coupled to a 10% decrease in BMD observed in the palmar aspect of both condyles. These components of variation matched the variations seen in the first three components of the shape-only and BMD only PCA models.

**Fig 3:**
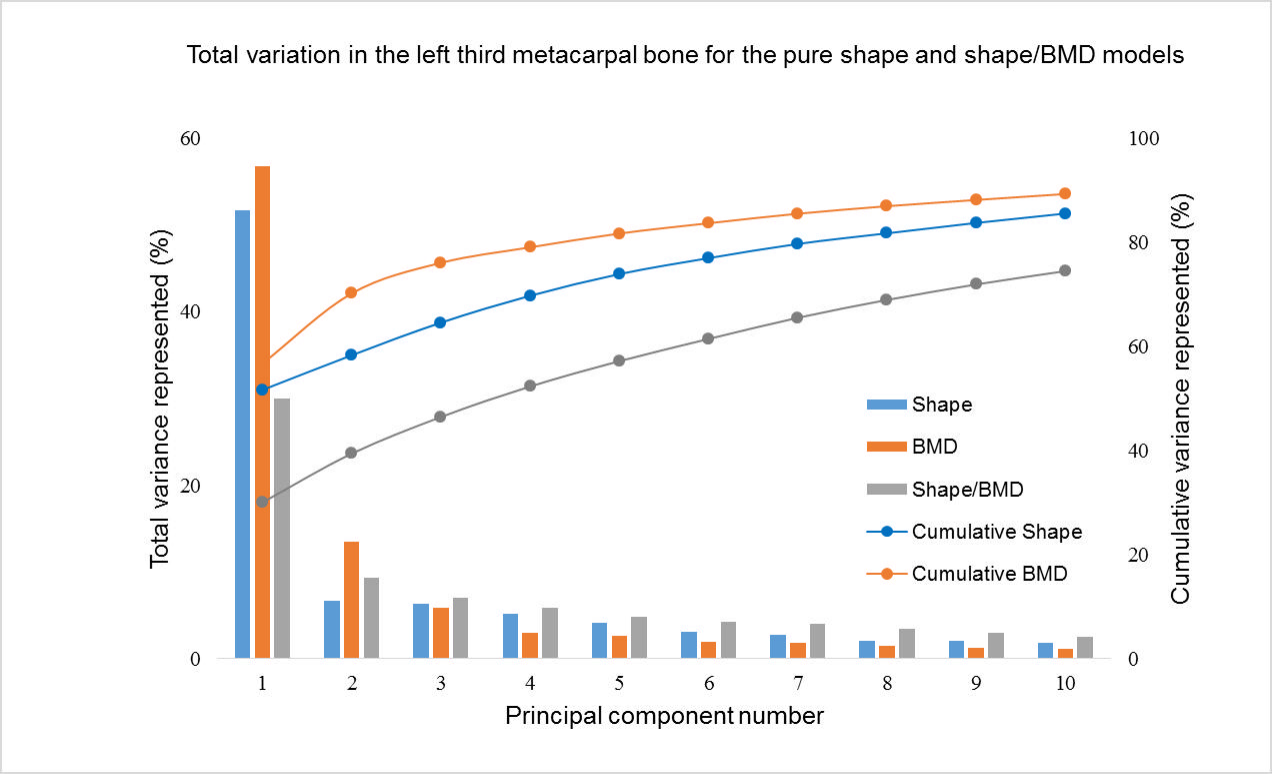
Absolute and cumulative variation for the principal component analysis on shape, BMD and combined shape/BMD models.

Population variance accounted for by subsequent components decreases considerably (Fig 3), suggesting that further components were not able to be uniquely defined from random noise.

### Leave-one-out analysis

**Fig 4:**
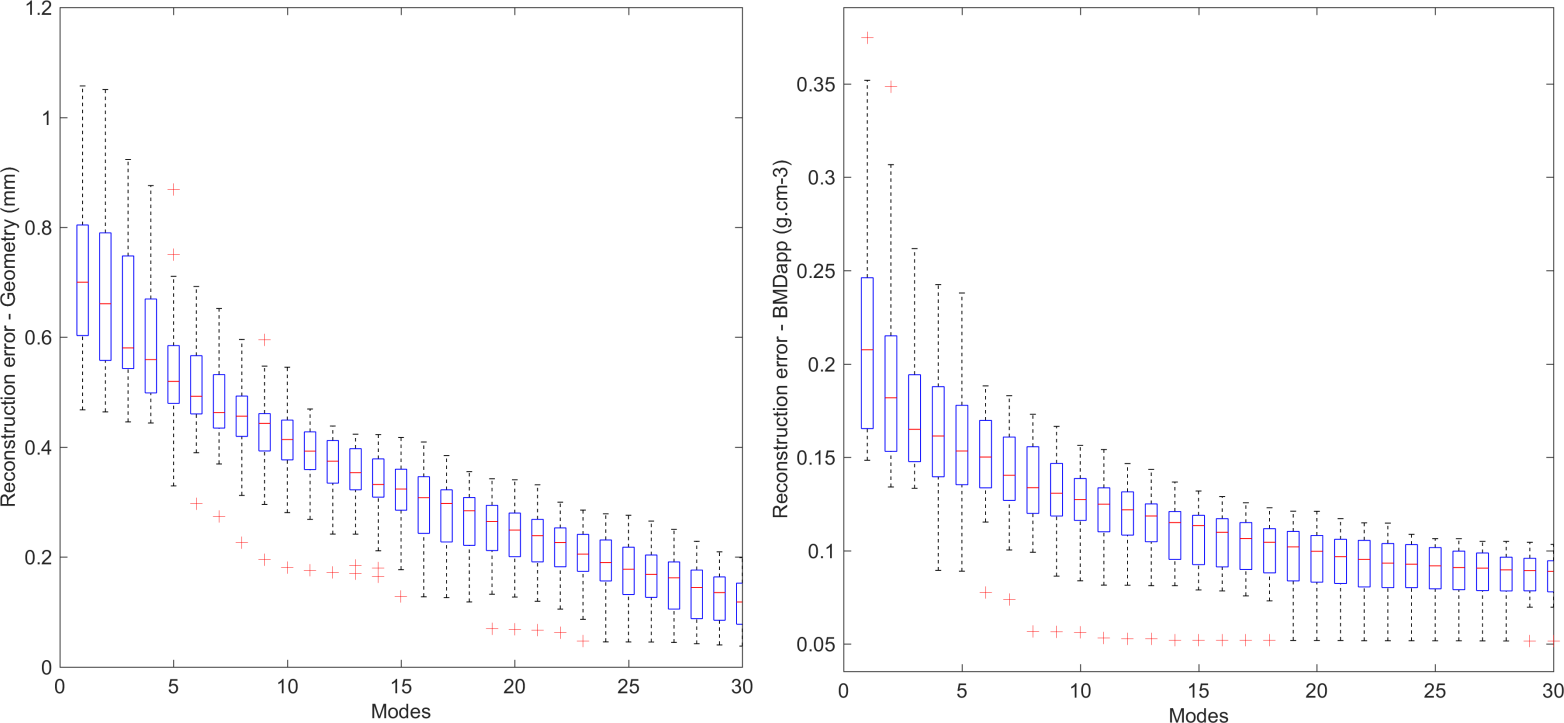
Reconstruction error of geometry and apparent BMD with increasing principal components.

A leave-one-out analysis was performed to assess the quality of the shape model and whether the size of the training set was sufficient. As shown in Fig 4, the model required 15 components to reduce the geometric RMS error to the voxel resolution (0.3 mm), and the BMD RMS to within 0.1 g.cm^-3^. 75% of the total variation was contained within the first ten components.

## Discussion

The first objective of this study was to develop a statistical shape model of the equine MC3 to describe morphology and subchondral BMD variation across a random population of 40 Thoroughbred horses. The first ten components of the statistical shape model described 75% of the total variation in correlated geometry and BMD. The leave-one-out analysis demonstrated that the model was able to successfully capture training set variability. The second objective of this study was to use this shape model to investigate whether the correlation of shape and bone mineral density shown in the model reflected existing observations of epiphyseal structure.

When assessing the statistical differences in shape and sub-chondral BMD distribution, it should be noted that gross pathological examinations were not performed on the specimens to identify existing pathology such as osteoarthritis.

The first principal component, accounting for 30% of the total variation, described an increase in overall size of the MC3 distal epiphysis, consistent with other statistical shape models describing bones morphology [23–25, 30, 37]. Size variation in the MC3 diaphysis with exercise has been quantified for young horses in initial training [19, 38]. However, these studies did not calculate scaling of the MC3 epiphysis. This result tells us that overall scaling of the bone was the biggest principal component, as opposed to any specific or localized change in shape. Since we did not have the height or weight of the horses in this study, it was not possible to determine the relationship between bone size and horse size.

In the current study, a qualitative examination of the size increase showed a more obvious change proximal to the circular curvature of the distal epiphysis. Similarly, size increase due to cortical thickening occurs mainly on the dorsal aspect of the MC3 diaphysis [19, 38]. The statistical shape model produced correlated bone mineral density changes, which showed that larger bones had higher BMD on the disto-palmar and dorso-proximal surfaces. Previous research has shown trained horses have increased BMD in these regions accompanied by diaphyseal size increase compared to untrained control horses [19]. The size variation seen in the statistical shape model here could be accounted by variable levels of training in this random sample of horses. Unfortunately, a complete, quantitative measure of training workload of these horses was not available, so this finding remains speculative.

The second component of variation accounted for 9% of the total variation, and can be qualitatively described as an increased convexity of the sagittal ridge at the dorsal junction of the epiphysis and the metaphysis. This shape change was coupled to increased BMD in that region. Previous research showed that low intensity initial training in 2 year old horses causes increased periosteal bone growth on the dorsal diaphysis. The same study observed increased BMD at the junction of the epiphysis and the metaphysis [19]. This region is where the proximo-dorsal aspect of the proximal phalangeal bone articulates with the third metacarpal bone at the stance phase of the gallop [1].

The third component of variation, which accounted for 7% of the total variation, showed a 10% increase in lateral condylar surface area relative to medial condylar area. As the lateral condyle increased in size relative to the medial condyle, the sub-chondral BMD at both dorsal condyles decreased. A study by Kawcak [15] showed that reduced lateral condylar area relative to medial condylar area was linked to condylar fracture. An increase in the An important limitation in this study was the lack of knowledge of the history of the horses. Age, sex and racing history could only be traced for less than 50% of the specimens. Therefore, the assumption that the sample contains a representative range of size, shape and exercise history may not be valid. The specimens with known history were predominantly female horses aged between 3 and 10 years. To add future value to this study, more specimens could be added to the modelling pipeline so that classification could be applied on the basis of age, sex, and racing history. Knowledge of the load history would add confidence to the form-function relationships, especially in very young horses.

surface area of bone can distribute the load over a wider area and lead to a decrease in the mechanical stress at the surface [39]. However, the statistical shape model in this study did not show increased asymmetry of shape to be linked to increased asymmetry of surface BMD.

The influence of these three components provides an insight into how this set of metacarpal bones varied. However, it is important to note that these components are coupled with other components of variation and will never occur in isolation. Thus, the shape and BMD of each metacarpal bone will be the product of the combined effect of a number of components, which may result in the features observed being cancelled out or exaggerated.

## Conclusions

The statistical shape analysis presented here produced a compact description of 3-D shape and material variations for the equine third metacarpal distal epiphysis. This is the first study to show shape variations in a sample population of MC3 bones, and to relate these shape changes to subchondral BMD. Because of the complexity of the shape, and the lack of easily identifiable landmarks, previous morphological observations of the MC3 bone epiphysis are sparse. This model builds on previous observations, and can be used to rapidly generate finite element models of the bones and joints for further structural analysis. A finite element simulation would show how the principal components of shape and BMD variation affect stress distribution through the bone, and possibly risk of fracture.

## Supporting information

S1 Table The first three principal components of variation for the correlated shape and bone mineral density PCA on the third metacarpal bone. (JPG)
