# Supplementary figures and images for: Statistical modeling of the equine third metacarpal bone incorporating morphology and bone mineral density
Source: PLoS One. 2018 Jun 6;13(6):e0194406. doi: 10.1371/journal.pone.0194406 (PMC5991359; doi:10.1371/journal.pone.0194406)

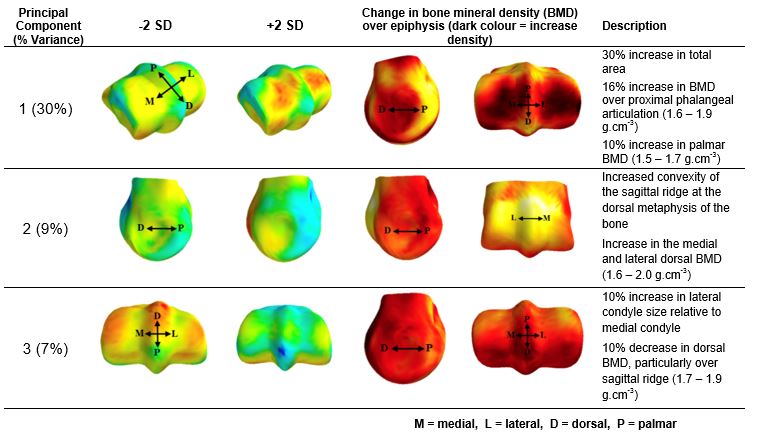

Supplement: S1 Table — (JPG) [file pone.0194406.s001.JPG]
